# The results of clinician-focused implementation strategies on uptake and outcomes of Measurement-Based Care (MBC) in general mental health care

**DOI:** 10.1186/s12913-023-09343-5

**Published:** 2023-04-01

**Authors:** Maartje A. M. S. van Sonsbeek, Giel J. M. Hutschemaekers, Jan W. Veerman, Ad Vermulst, Bea G. Tiemens

**Affiliations:** 1 Pro Persona Research, Pro Persona, Postbus 27, 6870 AA Renkum, The Netherlands; 2 Indigo Gelderland, Indigo Service Organisatie BV, Utrecht, The Netherlands; 3 Behavioural Science Institute, Radboud University, Nijmegen, The Netherlands; 4 GGZ (Mental Health Care) Oost Brabant, Boekel, The Netherlands

**Keywords:** Implementation, Hybrid design, Clinician-focused, Measurement-based care, General mental health care

## Abstract

**Background:**

Measurement-Based Care (MBC) is the routine administration of measures, clinicians’ review of the feedback and discussion of the feedback with their clients, and collaborative evaluation of the treatment plan. Although MBC is a promising way to improve outcomes in clinical practice, the implementation of MBC faces many barriers, and its uptake by clinicians is low. The purpose of this study was to investigate whether implementation strategies that were developed with clinicians and aimed at clinicians had an effect on (a) clinicians’ uptake of MBC and (b) clients’ outcomes of MBC.

**Methods:**

We used an effectiveness-implementation hybrid design based on Grol and Wensing’s implementation framework to assess the impact of clinician-focused implementation strategies on both clinicians’ uptake of MBC and outcomes obtained with MBC for clients in general mental health care. We hereby focused on the first and second parts of MBC, i.e., the administration of measures and use of feedback. Primary outcome measures were questionnaire completion rate and discussion of the feedback with clients. Secondary outcomes were treatment outcome, treatment length, and satisfaction with treatment.

**Results:**

There was a significant effect of the MBC implementation strategies on questionnaire completion rate (one part of clinicians’ uptake), but no significant effect on the amount of discussion of the feedback (the other part of clinicians’ uptake). Neither was there a significant effect on clients’ outcomes (treatment outcome, treatment length, and satisfaction with treatment). Due to various study limitations, the results should be viewed as exploratory.

**Conclusions:**

Establishing and sustaining MBC in real-world general mental health care is complex. This study helps to disentangle the effects of MBC implementation strategies on differential clinician uptake, but the effects of MBC implementation strategies on client outcomes need further examination.

## Background

Measurement-Based Care (MBC) is a promising intervention for improving outcomes in clinical practice. MBC is the routine administration of symptom, outcome or process measures, clinicians’ review of the feedback from these measures, clinicians’ discussion of the feedback with their clients, and collaborative evaluation of the treatment plan based on the feedback [1, 2]. This evidence-based practice is also referred to as Routine Outcome Monitoring (ROM) or Feedback-Informed Treatment (FIT).

Several studies have been conducted in adult mental-health care to assess the additional effects of MBC on treatment outcomes. The most comprehensive meta-analysis to date [2] reported a small positive effect of MBC on symptom reduction in both the full sample (*d* = 0.15) and a subsample of clients who were not progressing well through treatment (*d* = 0.17). The meta-analysis also reported a small favorable effect on dropout rates (*OR* = 1.20), but no effect on either treatment duration or the percentage of clients who had deteriorated by the end of treatment.

Little is known about potential mechanisms of action that underlie the effects of MBC. The Contextualized Feedback Intervention Theory (CFIT; [3]) assumes that when clinicians receive feedback, they compare the information from the feedback (e.g., the client’s progress in treatment so far) with the goal of the treatment (e.g., recovery). A discrepancy between the feedback and treatment goal causes clinicians to experience dissonance. This unpleasant feeling can encourage clinicians to generate increased effort toward achieving the goal. The Therapeutic Assessment Theory (TA; [4]) assumes that feedback from results to both the clinician and the client can improve the process of care because feedback enables clients to feel more involved in their own care. This promotes better communication and understanding of the client’s personal circumstances, allowing shared-decision making between the clinician and the client, enhancing the therapeutic alliance, increasing the client’s agreement with and adherence to the treatment by agreeing on shared goals, and the client’s satisfaction with the treatment [5, 6]. In turn, all of these factors can potentially improve client outcomes.

Although using MBC is considered good practice and can improve treatment results, the implementation of MBC has often been problematic [7] and uptake by clinicians low [8]. Problematic implementation of MBC has been attributed to barriers at different levels [1, 9, 10]: (a) the client (e.g., burden, time, concerns about a breach in confidentiality), (b) the clinician (e.g., attitudes, knowledge, self-efficacy, administrative burdens), (c) organization (e.g., training resources, leadership support, climate and culture), and (d) the system (e.g., accreditation, incentives, use of data by health insurers). Research across different countries indicates that typically fewer than 20% of clinicians employ MBC in their day-to-day work, only 5% of them use MBC every session, and 61.5% of them have never used MBC [11]. In the Netherlands, the same trends have been reported. Implementation is problematic [12] and use of MBC by clinicians in their daily practice is limited [13].

There are many different theories, models, and frameworks for implementing interventions in mental health care, each of which is based on different assumptions about human and organizational behavior [14]. However, the evidence for the validity of these theories, models, and frameworks is mixed and overall limited [15]. It can be concluded that the effective implementation of an intervention requires a systematic approach with good planning and adequate evaluation [16]. Grol and Wensing [17] offer a framework for such a systematic approach to implementation. It is based on both existing theories and models and practical experience. We used this framework in our study. The framework consists of different phases of tailored implementation: orientation, insight, acceptance, change, and maintenance. Each phase has a focus associated with it: (a) making clinicians aware of the innovation and getting them interested, (b) providing insight into their current way of working and the innovation, (c) getting clinicians motivated and intended to change, (d) implementing the innovation and confirmation of the usefulness, and (e) integration in existing routines within the organization. Within each phase, different implementation strategies can be used.

Grol and Wensing [17] provide suggestions for implementation strategies. In addition, the aforementioned research on barriers to MBC implementation has suggested complementary strategies for improving MBC implementation [9]. Multifaceted or blended strategies, which involve techniques for facilitating adoption, implementation, and sustainment, are needed [1]. These strategies include using relevant and valid questionnaires; using measurement feedback systems; leveraging local champions and opinion leaders; forming implementation teams with representatives from all stakeholder groups; having good instructional materials, clinician training, ongoing consultation and supervision; adaptation and integration of the innovation within the organization; and generating incentives [1, 9, 17]. Also, the effective and sustainable use of MBC might require systematic efforts over extended periods of time [18].

Few studies have evaluated the effects of MBC implementation [19]. In adult mental-health care, we found only two uncontrolled case studies [20, 21], two studies involving a national network [22, 23], one RCT [24], one cluster-randomized trial [25], and one mixed-methods study [26] that examined the effects of a specific implementation period. The implementation strategies in these studies involved education, asking or requiring clinicians to use MBC with all of their clients, the introduction of a digital MBC system with automated feedback, regular training of clinicians in MBC, clinical guidelines, and regular coaching or supervision to discuss clinical cases. On the one hand, these studies showed high rates of consistency in the use of the measures, high rates of compliance with the MBC procedure, and significant improvements in client retention, treatment outcomes, and expected client outcomes over the course of time [20–22, 24, 26]. Also, the impact of MBC increased across time. Brattland et al. [24] reported little effect of MBC in the first and second years of its use, but by the fourth year clients were two and one-half times as likely to improve when their clinicians used MBC compared to using treatment as usual. On the other hand, the studies reported a lack of effect of MBC implementation on full MBC fidelity [25], a low rate of discussion of the feedback with clients [22] and a remaning low use of MBC [23]. However, in most studies implementation and use of MBC seem to be used interchangeably. As a result, research on the implementation of MBC (which focuses on clinicians’ uptake) and clinical research on the effects of MBC (which focuses on clinical outcomes) seem to be intertwined. As far as we are aware, the separate effects of MBC implementation strategies on clinicians’ uptake and clients’ outcomes have hardly been examined.

Hybrid designs that simultaneously provide information on implementation processes and clients’ outcomes are needed. Three types of hybrid deigns have been defined [27]: (I) primary testing the effects of a clinical intervention on relevant outcomes and secondary observation and gathering of information on treatment implementation; (II) simultaneous testing the clinical intervention and the feasibility and potential utility of an implementation strategy; and (III) primary testing the effects of an implementation strategy and secondary assessment of clinical outcomes associated with the implementation strategy. By using a Type III hybrid design, it might be possible to disentangle the effects of MBC implementation strategies on clinicians’ uptake from clients’ outcomes. This could lead to better understanding of whether and how MBC implementation strategies affect clinicians and clients, and what needs to be changed to achieve better results.

In this study, we used a Type III hybrid design in a large general mental health care organization for adults with clinics throughout the Netherlands. We based our design on Grol and Wensing’s [17] implementation framework. Because the framework aims to optimize clients’ outcomes by improving how clinicians deliver their treatment, we call the strategies used within each phase *clinician-focused* strategies. Consistent with the framework, we divided the clinician-focused implementation strategies into different phases (three implementation phases and one follow-up phase). Furthermore, we shifted the emphasis of the phases from general to specific (i.e., from generic to clinic-focused to individual-focused). The clinician-focused implementation strategies were outlined before the implementation period began, but the strategies were refined during the implementation period, and additional strategies were added when results were found to be lagging.

The goals of the study were to investigate whether implementation strategies that were developed with clinicians and aimed at clinicians had an effect on (a) clinicians’ uptake of MBC and (b) clients’ outcomes of MBC. Clinicians’ uptake of MBC was the primary outcome measure and was operationalized as the questionnaire completion rate (i.e., the proportion of clients who completed the questionnaires during their treatment) and the extent to which the feedback had been discussed with the clients (as reported by the clients within the questionnaires). Clients’ outcomes of MBC was the secondary outcome measure and it was operationalized as treatment outcome (change in symptoms), treatment length (in days), and clients’ satisfaction with their treatment (as rated in the questionnaires). We expected that the application of clinician-focused implementation strategies would lead to (a) a higher rate of questionnaire completion and more discussion of the feedback by the clinicians, and (b) better treatment outcomes, a shorter length of the treatment, and clients’ greater satisfaction with treatment.

## Methods

The study adhered to the Standards for Reporting Implementation Studies (StaRI checklist [28]) and the Transparent Reporting of Evaluations with Nonrandomized Designs (TREND checklist; [29]).

### Design

We examined the results of our clinician-focused implementation strategies on both uptake of and outcomes from MBC by using an effectiveness-implementation Type III hybrid design, which was based on Grol and Wensing’s [17] implementation framework. The study was presented to the clinicians as a way to improve MBC and thereby the quality of the care they provided to their clients. It was conducted in a large organization for general mental health care in the Netherlands, which has clinics in several different regions. The clinician-focused implementation strategies were applied within one region of this organization (Implementation Group). The other regions did not receive any additional implementation support and were combined to form the Control Group.

### Context

Since 2011, mental health care organizations in the Netherlands have been required to collect outcome data for benchmarking on a national level. In January 2014, a distinction was made between general mental health care (for mild problems) and specialized mental health care (for complex problems). For the general mental health care, this resulted in changes in the organizational structures, the form of treatment, and the way of working. As a result, questions arose about how MBC could become a structural part of treatment in general mental health care and what additional effects MBC has in general mental health care. A need emerged for both improvements in MBC implementation and research on the effectiveness of MBC. To meet this need, the present study was initiated.

General mental health care organizations offer treatment to clients with common psychological disorders, which are too complex for the general practitioner to treat but for which specialist mental healthcare is too intensive. Examples of psychological disorders that are treated within general mental health care are: neurodevelopmental disorders, depressive disorders, anxiety disorders, and trauma- and stressor-related disorders. The large general mental health care organization in which the study was conducted offers four types of treatment: Short (up to five sessions), Middle (up to eight sessions), Intensive (up to twelve sessions), and Resilience (up to twelve sessions a year). The Short and Middle treatments are provided to clients with mild to moderately severe symptoms. They focus on improvements in the personal style of dealing with these symptoms. The Intensive treatments are provided to clients with moderate-to-severe psychological symptoms. Hereby, treatment is offered according to practice guidelines and focuses on symptom reduction. The Resilience treatments are provided to clients with a serious mental illness who need long-term support. These treatments focus on recovery instead of improvement. This study focused on clients who were receiving a Short, Middle, or Intensive treatment. Because clients in the Resilience category were receiving a different form of treatment, they completed a different kind of measures, and their treatment was by definition long-term, the Resilience treatments were excluded (*N* = 18,137 treatments).

The Short, Middle, and Intensive treatments were provided at clinics in seven regions throughout the Netherlands. All of the clinics were familiar with MBC, but the degree of implementation differed considerably among the clinics. However, one region in the eastern part of the Netherlands was strongly focused on MBC. Several clinic managers in this region expressed an explicit desire to improve the implementation of MBC, some of the clinicians had received training in MBC, and some of them were experimenting with MBC in their treatments. In addition, this region facilitated the research reported in the current paper on the implementation of MBC. Accordingly, the clinician-focused implementation strategies were applied in the clinics in this region. The four clinics in the region together formed the Implementation Group. The other regions were merged to form the Control Group. However, two regions (which provided 4,157 treatments, or 7% of total number of treatments) were excluded because these regions were not part of the organization during the entire implementation period. The four remaining regions, which were spread throughout the Netherlands, included eight clinics.

The clinician-focused implementation strategies were provided to all of the clinicians in the Implementation Group. There was a total of 44 clinicians at the start of the implementation period in 2015 and 42 clinicians at the end of the implementation period in 2018. However, due to high staff turnover, only 19 clinicians worked at the organization during the entire implementation period. At the start and end of the implementation period, there were no significant differences in the proportion of female clinicians (65.9%, *n* = 29 versus 64.3%, *n* = 27) nor in the primary discipline of the clinicians: psychologists (77.3%, *n* = 34 versus 69.0%, *n* = 29), nurses (15.9%, *n* = 7 versus 19.0%, *n* = 8), and psychiatrists (6.8%, *n* = 3 versus 19.0%, *n* = 8).

### Procedure

As part of routine care, the general mental health care organization in which the study was conducted had agreed in principle that its clients would be invited to complete the MBC questionnaires before their intake, after every third or fourth session during their treatment (depending on the type of treatment), and at the end of their treatment. However, the realization of this agreement lay within each region and no formal arrangements were in place regarding the number of measurements, actions that would be taken if clients did not respond to the request, nor how feedback would be discussed with the clients. Therefore, the actual frequency with which clients were invited to complete the MBC questionnaires varied across the different regions. In most of the regions, clients were invited to complete the questionnaires only at the beginning and the end of their treatment. Some individual clinicians, who were spread across different regions, encouraged their clients to also complete the questionnaires once or twice during their treatment. However, as far as we are aware, none of the clinicians invited their clients manually to complete the questionnaires at every treatment session.

As part of the clinician-focused implementation strategies, structural agreements were reached about the number and timing of the assessments for the clients in the Implementation Group. Because we aimed to maximize the likelihood that clinicians would use MBC, but the clinicians were not in favor of measuring at every session, we chose a moderate MBC frequency. Clients were automatically invited by an email sent from the measurement feedback system to complete the measures before intake, after every third or fourth session during treatment (depending on the type of treatment), and after the last session (or manually before the last session).

Clients in both groups were asked to complete (a) six demographic questions about marital status, educational level, household composition, and their employment situation, (b) five questions about hinderance and absence from work because of health problems, (c) the Outcome Questionnaire-45 (OQ-45, [30, 31], and (d) the EuroQol-5D [32] at every assessment. After the last session, clients were also asked to complete the Consumer Quality Index (CQI, [33, 34]. No other questionnaires were used as either MBC or as outcome measures. When clients were referred repeatedly to the general mental health care organization or received multiple treatments, they underwent the same procedure for each new treatment. This was the case for 5,375 (9.7%) clients.

After the questionnaires had been completed, the measurement feedback system automatically scored the questionnaires and generated a feedback report. Just as for the frequency of the MBC measurements, this report varied among the different regions. For the Implementation Group, the feedback report consisted of a written and graphic summary of all of the results, which were compared with previous scores, norms, and cut-off scores when applicable. The feedback report was accessible directly after the questionnaires had been completed in the measurement feedback system, and was transmitted to the electronic health record one day after the questionnaires had been completed. Prior to the study, clinicians were instructed to discuss the feedback reports with their clients. Clinicians received additional information and training during the study (see Phase 1 and Phase 2). For the Control Group, no instruction or training was provided.

### Implementation strategies

Consistent with Grol and Wensing’s framework [17], we developed different phases of the MBC implementation, each with different goals and different strategies. The phases were: (1) orientation and insight, (2) acceptance, (3) change, and (4) maintenance. The accompanying goals were: (1) to promote awareness, stimulate involvement, create understanding, and develop insight into own routines, (2) to develop a positive attitude toward change and create positive intensions to change, (3) to change and confirm the value of the change, and (4) to integrate changes in the routines and the organization. Furthermore, we shifted the focus of the phases from general to specific (generic, clinic-focused, and individual-focused). We selected the implementation strategies based on a combination of the Effective Practice and Organisation of Care taxonomy [35], Thorsen and Mäkelä’s [36] additional descriptions, and the applicability of the strategies to MBC and the clinicians within our general mental health care organization. We outlined the implementation strategies before the start of the implementation period, and we allocated the strategies to the different phases based on their focus (i.e., for clinicians in general, teams, or individuals). We further adapted implementation strategies in consultation with the clinicians in the MBC implementation team (see below) based on their experiences. Table 1 provides an overview of the operationalization of the implementation strategies. The implementation strategies were applied from February 2015 to January 2018 and with a follow-up that continued through December 2018.

**Table 1:** Operationalization of implementation strategies

Phase	Corresponding phase Grol and Wensing	Goal(s)	Time	Action	Actor(s)	Receiver(s)	Dose	Outcomes
1. General MBC strategies	Orientation and insight	Gain insight, outline possibilities for improvement, and get clinicians interested	February 2015 – February 2016	Delivering a global presentation	Principal researcher	All clinicians from organization	Once on internal training day	Primary: questionnaire completion rate discussion of feedback Secondary: treatment outcome treatment length satisfaction with treatment
				Forming an MBC implementation team	One clinician from each clinic, one manager, a highly experienced MBC researcher, and principal researcher	All clinicians from organization	Continuous with monthly meetings	
				Holding MBC theme meeting per clinic	MBC implementation team member from particular clinic and principal researcher	All clinicians within particular clinic	Once during team meeting	
				Providing monthly reports	Principal researcher	All clinicians from organization	Monthly by email	
2. Clinic-focused strategies	Acceptance	Gain acceptance of implementation strategies and motivate clinicians to change	March 2016 – March 2017	Continuation of MBC implementation team	Clinician from each clinic, manager, highly experienced MBC researcher, and principal researcher	All clinicians from organization	Continuous with monthly meetings	
				Continuation of monthly reports	Principal researcher	All clinicians from organization	Monthly by email	
				Training in MBC case consultation	Principal researcher	All clinicians within particular clinic	One hour per clinic	
				MBC case consultation	Clinician and colleague	Clinician and colleague	For every not-on-track client	
				Booster session in MBC case consultation	Principal researcher	All clinicians within particular clinic	One hour per clinic	
3. Individual-focused strategies	Change	Encourage clinicians who lag behind to improve	April 2017 – December 2017	MBC implementation team per clinic	Member of general MBC implementation team, clinic manager, and local champion	All clinicians within particular clinic	Continuous with regular meetings	
				Individual-focused monthly reports	Principal researcher	All clinicians from organization	Monthly by email	
				One-on-one appointments	MBC implementation team member from particular clinic/manager	Specific clinician	When questionnaire completion rate was below 50%	
4. Follow-up	Maintenance	Integration into routines and the organization	January 2018 – December 2018	Continuation of MBC implementation team per clinic	Member of general MBC implementation team, clinic manager, and local champion	All clinicians within particular clinic	Continuous with regular meetings	

#### Phase 1: General MBC strategies (February 2015 – February 2016)

The main purpose of Phase 1 was to gain insight into the importance and the current state of MBC within the clinics, to outline possibilities for improving MBC, and to get clinicians interested to improve their use of MBC. The main implementation strategies used in this phase were (1) delivering a global presentation, (2) forming an MBC implementation team, (3) holding an MBC theme meeting in each clinic, and (4) providing monthly reports.

The starting point was the global presentation by the principal researcher. Because the presentation was given on an internal training day, most of the managers and clinicians attended. During the presentation, the current status of MBC and the study were outlined and the clinicians were invited to ask questions and give feedback.

The MBC implementation team comprised seven members: one clinician from each clinic, one manager, a highly experienced MBC researcher, and the principal researcher (who is both a mental health care psychologist in one of the clinics and an MBC researcher). The members were self-selected on the basis of conversations and their positive attitude toward and interest in MBC. The premise was that the members of the MBC implementation team would serve as local champions and hence opinion leaders. The MBC implementation team was responsible for optimizing the conditions under which MBC would be delivered, developing and executing the implementation strategies, and identifying barriers and finding solutions. The MBC implementation team had monthly meetings and they set out actions on an ongoing basis. Examples of actions that the team took were as follows: they developed enhancements in the feedback report, developed and disseminated the MBC instructional materials, provided the clinicians with MBC cards with reminders and an action plan, and insured a continuing focus on MBC during clinic meetings.

Each member of the MBC implementation team started by organizing a clinic-specific MBC theme meeting in his or her own clinic. At this meeting, the member of the MBC implementation team and the principal researcher gave more information about the study and the MBC implementation team, and discussed practical aspects of MBC (e.g., where to find the feedback report, how to interpret the feedback report, and how to discuss the feedback with the client).

The monthly report was produced by the principal researcher and indicated the questionnaire completion rates in both the preceding month and over the course over time. The report was sent to the members of the MBC implementation team, and the managers and leading experts of each location. In the accompanying email, the cross-clinic conclusions were summarized, and clinics with more than 80% of the questionnaires completed at the start of treatment were congratulated and given a pie and an inspirational card.

#### Phase 2: Clinic-focused strategies (March 2016 – March 2017)

The main purpose of Phase 2 was to gain each clinic’s acceptance of the implementation strategies and foster the clinicians’ motivation to change their behaviour. The main implementation strategies used in this phase were introduction of and training in MBC case consultation. Additionally, the members of the MBC implementation team continued with their activities.

During the MBC case consultation, the clinician was supposed to discuss with a colleague the feedback of those clients who were not improving (the *not-on-track* clients) while using a standardized format for case consultation. The idea was that the clinician would choose a colleague with personal relevance for him or her and whose advice he or she would trust. The case consultation format included questions about how to explain negative results, implications for the treatment, and advice from the colleague. The format was designed to be completed within 15 min and it was integrated into the client’s electronic health record. Thus, through MBC case consultation, both treatments could be improved and the clinician’s use of feedback could be monitored.

Before starting with the MBC case consultation, the principal researcher provided each clinic with a one-hour training session. The training consisted of a repetition of general and practical information about MBC, explanation of the MBC case consultation and the case consultation format, practice in conducting an MBC case consultation, and discussion of the clinicians’ experiences. After the initial training, the monthly reports were supplemented with an overview of the *not-on-track* clients and the proportion of case consultation in each clinic. Also, in the MBC feedback report, a reminder was added to discuss within the MBC case consultation when a client was not-on-track. After four months, a one-hour, clinic-tailored booster session was provided in which the number of MBC case consultation to date were discussed; barriers were identified and resolved where possible; and clinicians were invited to suggest improvements by defining their own MBC action points. Between and after the training sessions, the MBC implementation team was available for additional support.

#### Phase 3: Individual-focused strategies (April 2017 – December 2017)

The main purpose of Phase 3 was to encourage clinicians who lagged behind to improve. The main implementation strategies included: (1) having an MBC implementation team at each clinic, (2) individual-focused monthly reports with additional rewards for achievements, and (3) one-on-one MBC appointments.

The MBC implementation team at each clinic included the member of the general MBC implementation team, the clinic manager, and a local champion of MBC. The aim of the local MBC implementation team was to get more clinicians involved, to specify and expand the implementation strategies, and to find solutions for local barriers. The local MBC implementation team met regularly (although the frequency of the meetings varied by clinics), and the team set out actions on an ongoing basis.

The monthly report was supplemented by results from each individual clinician. Clinics received a pie when they conducted case consultations for more than 50% of the *not-on-track* clients, and individual clinicians received flowers when they met the criterion that was set by the local MBC implementation team (e.g., case consultation for at least 80% of their *not-on-track* clients).

To support clinicians who were struggling with MBC, each clinician with a questionnaire completion rate below 50% was offered a one-on-one meeting with a member of the MBC implementation team. During this meeting, possible obstacles were identified and solutions were considered. When the clinician was unable to improve the questionnaire completion rate during the following months, the clinician was invited to a meeting with the manager to consider additional steps for improving MBC (e.g., coaching or supervision).

#### Phase 4: Follow-up (January – December 2018)

In the fourth phase, we monitored whether MBC had been integrated into the clinicians’ day-to-day practice and whether the changes were being maintained in the clinics. The coordination for maintaining and further improving MBC was the responsibility of the local MBC implementation team. This implied that the production and distribution of reports, the content and focus of the reports, possible rewards for achievements, the planning of one-on-one appointments, and any new implementation strategies were determined by the local MBC implementation team. The members of the general MBC implementation team, including the principal investigator, remained available to foresee possible obstacles, to think of ways to overcome them, and to facilitate the general maintenance of MBC. No additional implementation strategies were conducted during this phase.

### Study variables

#### Background variables

Baseline characteristics of clients in both the Implementation Group and the Control Group were collected at the start of treatment. These characteristics were: age, gender, education, household composition, marital status, diagnosis, and total difficulties score on the Outcome Questionnaire-45 [37]; see below).

#### Uptake by clinicians

The first goal of this study was to investigate whether the implementation strategies that were developed with clinicians and aimed at clinicians improved the clinicians’ uptake of MBC. Thus, the primary outcome measures were questionnaire completion rate and extent to which the feedback had been discussed with the clients.

Because routine administration of the measures is a requirement in MBC, we defined questionnaire completion rate as the proportion of clients who had completed the questionnaires at the beginning of treatment, at least once during treatment, and at the end of treatment. This number was compared to the total number of clients who were referred for general mental health care at the organisation within a specific six-month period. In addition, we considered questionnaire completion as valid only if the questionnaires had been completed within 30 days of the beginning or the end of the treatment (i.e., the date of the first or the last treatment session). The questionnaire completion rate was derived from the electronic health record system.

The discussion of feedback was the extent to which the clinicians discussed the feedback reports with their clients. This was based on clients’ answers to Question 12 on the Consumer Quality Index (CQI) for Mental Health care and Addiction Services, Version 1.0 [34], which was administered at the end of treatment. Question 12 on the CQI states, “Before and perhaps during treatment, you or your clinician(s) completed one or more measures about how you were doing at that time. Were the results discussed with you?” The response options were: *no, not at all (1)*; *a little bit (2)*; *partly (3)*; *largely (4)*; *yes, completely (5)*, and *not applicable (no lists completed)*.

#### Client outcomes

The second goal of this study was to investigate whether the implementation strategies that were developed with clinicians and aimed at clinicians improved clients’ outcomes of MBC. Therefore, secondary outcome measures were treatment outcome, treatment length, and satisfaction with treatment.

Treatment outcome was the degree of change in symptoms and functioning as measured by the Outcome Questionnaire-45 (OQ-45, [37]. Treatment outcome was, therefore, available only for clients who completed the questionnaires at the start and end of treatment. The OQ-45 consists of 45 items, which are scored on a five-point scale ranging from *never (0)* to *nearly always (4)*. The total difficulties score (range: 0–180) reflects symptom distress, problems with interpersonal relationships and problems with the social role. Change was defined as the total score at the start of the treatment *minus* the total score at the end of the treatment. The Reliable Change Index (RCI; [38]) was used to determine whether the change score for each client was statistically significantly greater than the difference that could have occurred because of random measurement error alone. The RCI for the OQ-45 total score is 14, so a client must improve at least 14 points in order to show reliable change. The Dutch OQ-45 has been found to have adequate and similar psychometric properties as the original OQ-45 [30].

Treatment length was measured as the number of days between registration and deregistration at the general mental health care institution. Treatment length was derived from the electronic health record system.

Satisfaction with treatment was measured with Question 13 on the CQI: What rating do you give the treatment? Response options were *0* to *10*, where *0* means *very poor* and *10* means *excellent*. Only the clients who completed the questionnaires at the end of treatment could answer the question about satisfaction with treatment.

### Statistical analysis

#### Missing values

The percentage of missing values differed widely between the outcome measures that were derived from the electronic health record system (outcome questionnaire completion rate and treatment length) and the measurement feedback system (discussion of feedback, change in symptoms, and satisfaction with treatment). Because the outcome questionnaire completion rate signals whether or not each client completed the questionnaires at both the beginning of treatment, at least once during treatment, and at the end of treatment, this outcome measure could be determined for each client, and it had no missing values. The percentage of missing values for treatment length (in days) was 0.3. For discussion of feedback (CQI Question 12), change in symptoms (OQ-45 difference score), and satisfaction with treatment (CQI Question 13), the percentage of missing values was 89, 72, and 73, respectively.

Independent sample *t*-tests and chi-square tests were used to determine whether the non-missing cases differed from the missing cases on each of the seven background variables. Because of the large sample size, almost all of the tests showed significant results. To help us decide whether the significant results were meaningful, we used Cohen’s *d* (based on the *t*-test results) and *phi* coefficients (based on the chi-square test results) as effect sizes. Cohen’s *d* with effect sizes of 0.2 and *phi* coefficients with effect sizes of 0.1 are considered small effect sizes [39]. Cohen’s *d* varied between 0.02 and 0.15, and *phi* varied between 0.01 and 0.06. These very small effect sizes suggest that in spite of the significant differences, there are no substantial differences between the non-missing and missing cases. For this reason, we assumed that the group with non-missing values was representative of the entire group for each of the three variables.

#### Analysis

For the statistical analyses the nested structure of the dataset must be taken into account. Treatment episodes (or referrals) are nested within clients, clients are nested within clinicians and clinicians are nested within regions. However, the system from which our dataset is derived is client-based and does not include information about clinicians. Therefore, accounting for nesting within clinicians is not possible. To control for nesting of multiple treatment episodes within clients the COMPLEX procedure of the statistical software package Mplus version 7.4 was used [40]. In this approach standard errors of parameter estimates are computed by taken into account the dependency of the data caused by multiple treatments. Controlling for nesting of clients within regions in this way is not adequate because the number of regions (one for the Implementation Group and four for the Control Group) is too low for complex or multilevel analysis. Instead, we used dummies to control for region in the Control Group (three dummies for four regions) only if it was possible with the used statistical analysis technique. Correcting for multiple treatment episodes and for region in the Control Group had little effect on the results. For the regression analyses we compared the uncorrected analyses results with the corrected ones. Test statistics and *p*-values changed with minor differences (sometimes a little bit higher and sometimes a little bit lower), resulting in the same conclusions.

We described baseline characteristics (seven variables) and tested differences between the Implementation and Control Group using regression analysis with group membership as predictor and each of the seven variables as outcome variable. For age and total difficulties score (OQ-45) linear regression was used and probit regression for the other (ordered categorical) variables. The COMPLEX procedure was used to control for nesting of multiple treatment episodes within clients. Controlling for region in the Control Group only was not possible. With the Wald test the estimated regression model is compared with a baseline model (regression coefficient equals zero) resulting in a Chi-square value with one degree of freedom.

For each outcome variable (questionnaire completion rate, discussion of feedback, change in symptoms, treatment length, and satisfaction with treatment) we plotted how the outcome variable changed over time for the Implementation and Control Group.

When an outcome variable showed both increases and decreases, we made a more detailed description in relation to the four phases of the implementation strategies. We tested differences in proportions between two subsequent half-year periods with z-tests for two independent proportions. Correction for multiple measurements and regions was not possible. Not only because the z-tests were calculated by hand, multiple measurements within clients between two subsequent half-year periods were negligible.

To estimate total change over time for the Implementation and Control Group we used regression analysis with time as independent variable and each of the outcome variables as dependent variable with the slope as estimate of change over time. For questionnaire completion rate we applied probit regression, for the other four outcome variables linear regression was used. For nesting of multiple treatment episodes within clients the correction approach COMPLEX in Mplus was used. To correct for nesting of clients within regions dummies were used in the Control Group. The probability to find significant slopes is high in large samples. Therefore, in addition we calculated effect size *f*^*2*^ with values of 0.02, 0.15 and 0.35 reflecting a small, medium and large effect size respectively [39].

To test the effect of implementation we compared the slope of the Implementation Group with the slope of the Control Group using z-tests for differences between two independent slopes [41]. To our knowledge effect sizes for differences between two slopes have not been developed yet.

The analyses were conducted using IBM SPSS statistics version 26 [42] and Mplus version 7.4 [40].

## Results

The final sample comprised 50,272 unique clients with a total of 55,647 treatment episodes. Thus, 5,375 clients (9.7%) were referred repeatedly to the general mental health care organization or received multiple treatments. Because our implementation strategies were focused on the clinician, treatment was the unit for analysis. The Implementation Group had provided 8,458 (15.2%) treatments and the Control Group 47,189 (84.8%) treatments.

The mean age of the clients was between 39 and 40 years (Implementation Group mean = 39.2 years, *sd* = 14.1, range = 19-to-95 years; Control Group mean = 39.7 years, *sd* = 13.6 years, range = 19-to-94 years), and 40.6% (Implementation Group) and 37.7% (Control Group) were male. The majority of the clients had completed secondary school (Implementation Group: 69.8%; Control Group: 67.5%), lived together (Implementation Group: 68.0%; Control Group: 69.8%), and were unmarried (Implementation Group: 55.5%; Control Group: 53.3%). The most common primary diagnoses were anxiety (Implementation Group: 42.1%; Control Group: 39.6%) and depressive disorders (Implementation Group: 36.7%; Control Group: 40.1%). The average OQ-45 total score at the start of the treatment was 79.7 (*sd* = 22.8, 86.3% of the clients scored ≥ the cut-off score of 55) for the Implementation Group and 80.4 (*sd* = 23.5, 86.4% of the clients scored ≥ the cut-off score of 55) for the Control Group. A full description of the baseline characteristics of the clients in the Implementation Group and the Control Group is presented in Table 2.

**Table 2:** Clients’ baseline characteristics

Baseline characteristics	Implementation group	Control group	Comparison Wald χ^2^-test	p
*n*	8,458	47,189		
Age, M *(sd)*	39.2 (14.1)	39.7 (13.6)	χ^2^(1)=9.04	.003
Male, *n* (*%*)	3,433 (40.6)	17,767 (37.7)	χ^2^(1)=21.60	.000
Highest education			χ^2^(1)=55.88	.000
Primary school, *n* (*%*)	512 (7.0)	1,129 (5.3)		
Secondary school, *n* (*%*)	5,106 (69.8)	14,281 (67.5)		
College or university, *n* (*%*)	1,694 (23.2)	5,762 (27.2)		
Household composition			χ^2^(1)=.55	.460
Alone *n* (*%*)	1,919 (26.2)	5,417 (25.5)		
Together *n* (*%*)	4,972 (68.0)	14,794 (69.8)		
Other *n* (*%*)	424 (5.8)	993 (4.7)		
Marital status			χ^2^(1)=18.04	.000
Unmarried *n* (*%*)	4,059 (55.5)	11,990 (53.3)		
Married *n* (*%*)	2,238 (32.0)	7,768 (34.5)		
Divorced *n* (*%*)	745 (10.2)	2,306 (10.3)		
Widow(er) *n* (*%*)	173 (2.4)	426 (1.9)		
Main diagnosis			χ^2^(1)=7.53	.006
Anxiety *n* (*%*)	3,248 (42.1)	15,829 (39.6)		
Depression *n* (*%*)	2,832 (36.7)	16,032 (40.1)		
Somatic Symptom *n* (*%*)	671 (8.7)	2,783 (7.0)		
Other *n* (*%*)	963 (12.5)	5,366 (13.5)		
OQ-45 total start, *M (sd)*	79.7 (22.8)	80.4 (23.5)	χ^2^(1)=4.79	.029

The baseline characteristics show significant differences between the Implementation and Control Group for all variables with the exception of household composition. Comparing the means (for age and OQ-45) or the percentages (for the other variables), the differences between both groups are very minimal. Despite the significant differences the results show that the baseline characteristics show minor differences between the two groups.

**Fig. 1:**
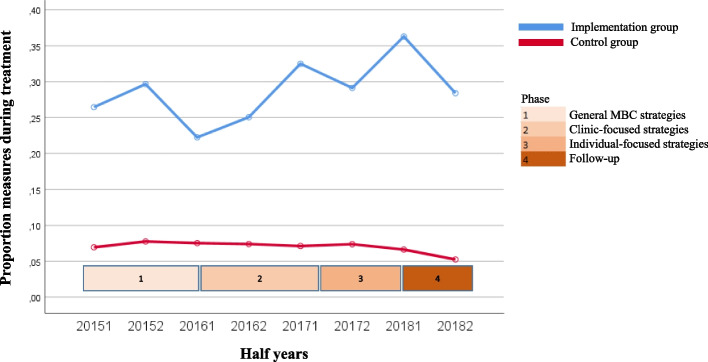
Questionnaire completion rate

**Fig. 2:**
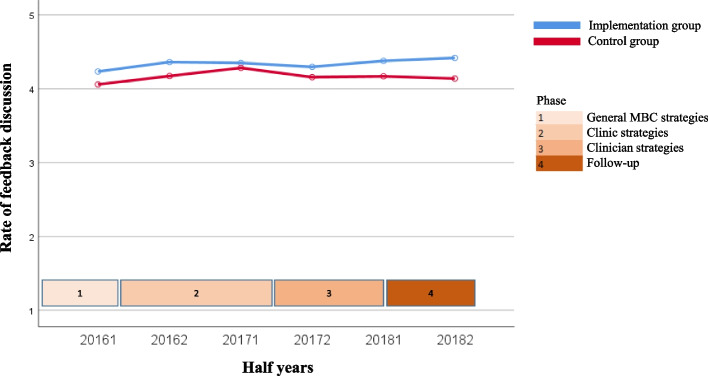
Discussion of feedback (In 2015, the question about discussion of the feedback was answered for only 10 treatments; 7 treatments within the Implementation group and 3 treatments in the Control Group). Therefore, the results from 2015 and the first half of 2016 were merged for the analysis)

**Fig. 3:**
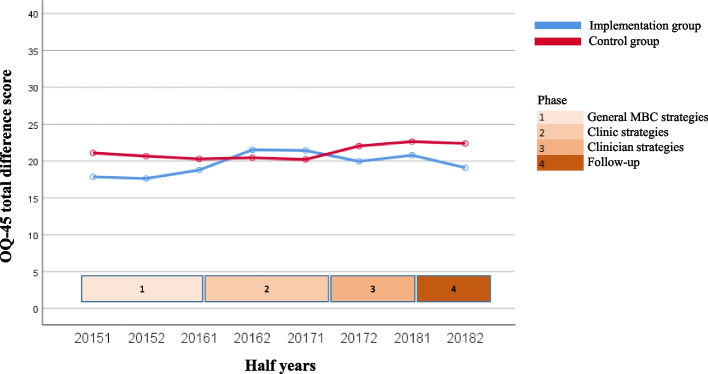
Change in symptoms

**Fig. 4:**
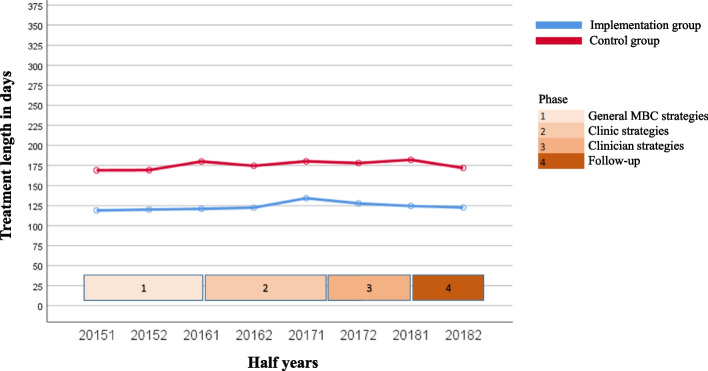
Treatment length

**Fig. 5:**
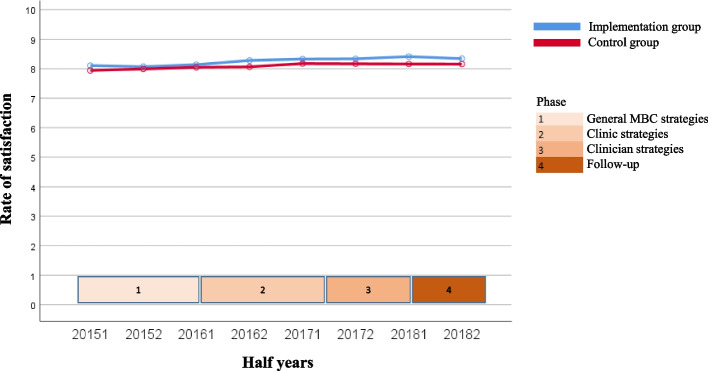
Satisfaction with treatment

The course over time for the variables for both the uptake of MBC by clinicians (questionnaire completion rate and degree of discussion of feedback) and client outcomes of MBC (degree of change in symptoms, treatment length, and satisfaction with treatment) are shown in Fig. 1, 2, 3, 4 and 5. Because the Implementation Group was already strongly focused on MBC, the baseline level for the questionnaire completion rate was higher for the Implementation Group (26%) than for the Control Group (8%). It should also be noted that for the Implementation Group the treatment length was lower (mean = 124.11 days, *sd* = 83.77) than for the Control Group (mean = 175.46 days; *sd* = 101.52). In addition, the clinicians and clients in the Implementation Group seem to have selected Short treatments more often (9.5%, *n* = 803) but Intensive treatments less often (53.1%, *n* = 4,487) than the clinicians and clients in the Control Group (5.2%, *n* = 2,461 versus 60.4%, *n* = 28,515, respectively).

For the questionnaire completion rate in the Implementation Group (see Fig. 1) a significant decrease in questionnaire completion rate in the second part of phase 1 (second half of 2015 to first half of 2016, *z* = -3.81, *p* = 0.000), a significant increase in the second part of phase 2 (second half of 2016 to first half of 2017, *z* = 3.97, *p* = 0.000) and phase 3 (second half of 2017 to first half of 2018, *z* = 3.53, *p* = 0.000), and a significant decrease in phase 4 (first half of 2018 to second half of 2018, *z* = -3.86, *p* = 0.000) was found. Overall, the questionnaire completion rate increased from 26 to 37% during the implementation phases, before almost dropping down to the baseline level during the follow-up phase (29%). The average questionnaire completion rate for the Implementation Group was 28.8%. The questionnaire completion rate in the Control Group was fairly stable and varied around 8% (the individual regions were 7.9%, 8.9%, 5.7%, and 7.5%, respectively).

The other outcome variables (Figs. 2, 3, 4 and 5) show a rather stable course over time. The mean rate of discussion of the feedback varied between 4 and 4.5 scale points for both groups (“results were largely discussed”; the Implementation Group varied from 4.23 to 4.41 and the Control Group varied from 4.39 to 4.66). The mean OQ-45 difference score varied around 20 points (Implementation Group: from 18.28 to 20.91 points; Control Group: from 20.28 to 22.57 points). The average OQ-45 total score at the end of treatment was 60.77 for the Implementation Group (*sd* = 25.80, 54.7% of the clients scored ≥ the cut-off score of 55, and 57.9% of the clients had an OQ difference score ≥ the RCI of 14 points), and 59.39 for the Control Group (*sd* = 26.64, 52.7% of the clients scored ≥ the cut-off score of 55, and 58.9% of the clients had an OQ difference score ≥ RCI of 14 points). The mean treatment length was an almost flat line around 125 days for the Implementation group (the mean treatment length was between 118.94 and 134.21 days) and 175 days for the Control Group (the mean treatment length was between 168.96 and 181.94 days). The same holds true for the rate of satisfaction (around 8 for both groups; Implementation Group: from 8.06 to 8.43 and Control Group: from 8.30 to 8.54).

Table 3 shows the results of the regression analyses with time as predictor and each of the outcome variables as dependent variable. Intercepts (b0) can be interpreted as the estimated starting level and regression coefficients (b1) as the mean change (increase or decrease) over time per year. All variables, other than discussion of feedback, showed a significant increase over time for both the Implementation and Control Group. The exception was questionnaire completion rate in the Control Group, for which a significant decrease was found (*z* = -4.09, *p* = 0.000). However, the effect sizes were very small (*f*^*2*^ ≤ 0.001).

**Table 3:** Results of regression analysis of outcome variables on time, controlling for nesting of multiple treatments within clients and for nesting of clients within regions

	Group	n treatments	b0	b1	beta	z	p	effect size (*f*^*2*^)	Differences between b1’s of both groups	
									Z	p
Questionnaire completion rate	Implementation	8,458	0.668	.052	.060	4.07	**.000**	.001	5.57	.**000**
	Control	47,189	1.347	-.033	-.037	-4.09	**.000**	.000		
Discussion of feedback	Implementation	2,428	4.230	.044	.028	1.33	.184	.000	-.48	.631
	Control	3,752	4.389	.067	.036	1.94	.052	.000		
Change in symptoms	Implementation	3,364	18.283	.657	.034	2.03	**.043**	.000	.22	.826
	Control	11,939	20.281	.574	.028	3.13	**.002**	.000		
Treatment length	Implementation	8,406	119.662	2.108	.029	2.65	**.008**	.000	-.26	.795
	Control	47,055	158.754	2.345	.026	5.72	**.000**	.000		
Satisfaction with treatment	Implementation	3,583	8.060	.093	.070	3.98	**.000**	.000	1.32	.187
	Control	11,641	8.300	.060	.042	4.41	**.000**	.000		

Only for questionnaire completion rate a significant difference was found between the slopes of the Implementation Group and the Control Group (*z* = 5.57, *p* = 0.000). The increase in questionnaire completion rate over time was significantly positive for the Implementation Group and significantly negative for the Control Group with very small effect sizes (*f*^*2*^ ≤ 0.001).

## Discussion

We used an effectiveness-implementation Type III hybrid design based on Grol and Wensing’s [17] implementation framework to investigate the effects of clinician-focused implementation strategies on both clinicians’ uptake and clients’ outcomes of MBC in general mental health care. We, therefore, focused on the first and second parts of MBC (administration of measures and the use of feedback). The results showed only a significant effect of the MBC implementation strategies on questionnaire completion rate (one indicant of clinicians’ uptake). During implementation, the rate at which questionnaires were completed in the Implementation Group increased from 26 to 37% before decreasing to almost 29% during follow-up. The questionnaire completion rate in the Control Group was stable at about 8%. Effects were found for neither the degree of discussion of feedback nor clients’ outcomes (treatment outcome, treatment length, nor satisfaction with treatment).

It is intriguing yet concerning that despite all the strategies we used, there was only an increase in the number of clients who completed the questionnaires. We expected that this would lead to more discussion of the feedback between clinicians and clients, and the benefits of using the feedback would increase both clients’ outcomes and clinicians’ integration of MBC into daily practice. However, such a self-enforcing loop [43, 44] never emerged because clinicians only stimulated more clients to complete the questionnaires but did not change their own behaviour (discussion of feedback with clients and colleagues). Explanations for our limited results and why the self-enforcing loop did not occur can be classified into several categories.

### Clinician factors

It seemed that the clinicians had an insufficient sense of urgency. People with a sense of urgency have a vision of why change is needed, feel that immediate change is needed, and are committed to change (e.g., [45]. This should instil the motivation to abandon current behavioral patterns and adopt new ones. There was urgency for MBC because many clients (up to 55%) do not progress or even deteriorate during treatment [46] and clinicians are unable to identify clients who are likely to deteriorate (e.g., [47]. Also, previous research has reported positive outcomes for clients when clinicians were self-selected to participate in MBC [48]. Through the combination of the urgency, the explicit desire of several clinic managers within the implementation region to improve MBC implementation, and our multifaceted implementation strategy with an increasing focus on the individual clinician, we anticipated that clinicians would be motivated to improve their use of MBC. However, our expectations did not come true. In addition, the clinicians’ encouragement of clients to complete the questionnaires seemed externally instead of internally motivated. Beginning in 2011, Dutch mental health care organizations received financial incentives for submitting MBC data to the mental health benchmark foundation (SBG), and health insurers used the results to establish purchasing policies [49]. Many clinicians had built up resistance against SBG, and consequently did only ‘what was required’ (achieve a sufficient questionnaire completion rate). This could explain the lack of requests for additional training, poor completion of the case consultation format, and MBC not being sufficiently integrated into daily practice to become a routine (e.g., [50]. However, we were not able to evaluate the impact of clinician factors and to account for nesting of the data within clinicians. This was because the system from which our dataset was derived does not include information about clinicians. Therefore, clinician factors might have influenced the results, and the results might have been different if we had been able to take the nesting of clients within clinicians into account.

### Implementation framework

It is possible that Grol and Wensing’s [17] framework did not provide sufficient guidance for implementing MBC in general mental health care. The effectiveness of a multifaceted implementation plan is determined by the effectiveness of the separate underlying strategies and their interaction [17]. Perhaps we (a) chose ineffective strategies for mental health care or for MBC, (b) combined too many strategies in too short a period of time, or (c) paid insufficient attention to specific clinicians’ or organization’s characteristics [51, 52].

### Study design

First, we used a quasi-experimental design to closely match the desire of several clinic managers within the implementation region to improve MBC implementation. The consequence of a quasi-experimental design is that it is more complex to find causes and effects due to the lack of randomization.

Second, we chose to administer the MBC questionnaires after every third or fourth session rather than at every session. Although we are aware that administering the MBC questionnaires at every session is recommended, the clinicians did not support the idea of session-by-session measurements and were unwilling to use MBC that frequently. Thus, we had to find a compromise between measuring as frequently as possible and getting clinicians on board for using MBC. However, the relatively low frequency might have prevented the use of MBC from becoming routine and habitual for the clinicians [53]. Indeed, the total questionnaire completion rate for the region of the Implementation Group was only 28.8% (*n* = 2439), and for the regions in the Control Group 7.9% (*n* = 372), 8.9% (*n* = 1232), 5.7% (*n* = 1405), and 7.5% (*n* = 307), respectively.

Third, clients were asked to complete a relatively long combination of questionnaires at every assessment (demographic questions, questions about absence from work, the OQ-45, and the EuroQol-5D). This may have hindered clients from completing the questionnaires and continuing to complete them regularly.

Fourth, we determined the extent to which the feedback had been discussed with clients by using a retrospective, single item, which was administered at the end of treatment as part of the MBC questionnaires instead of routinely after each session. Using only one question that is filled out by the clients at the end of treatment may be too limited as a measure of discussion of the feedback. This could have affected the results and limited the evaluation of specific implementation strategies.

Fifth, we had to exclude two regions (with 4,157 treatments, or 7% of the total number of treatments) because these regions were not a part of the organization during the entire implementation period. We compared clients' baseline characteristics in the Control Group (see Table 2) with those in the two excluded regions and found minor differences between the two groups. Although it thus seems likely that excluding the two regions had little impact on the data and the analysis, we cannot completely rule out the possibility that excluding the two regions affected the results of the study.

Sixth, there was an imbalance between the number of clinics in the Implementation Group and the Control Group. Because one region explicitly expressed a desire to improve the implementation of MBC, the four clinics in this region became the Implementation Group, and the other eight clinics (in four different regions) formed the Control Group. The population and practices in the eight clinics included in the Control Group may have varied more than those of the four clinics in the Implementation Group and might therefore have increased the variance and affected the results. For example, one clinic reported that 29% (*n* = 4027) of the clients had only a primary diagnosis and no secondary diagnosis, whereas another clinic reported a primary diagnosis for 100% (*n* = 4076) of its clients. Also, the highest education attained varied considerably among the clinics. At one clinic, only 3.0% (*n* = 18) of the clients reported that completing primary school was their highest level of education, and 46.4% (*n* = 276) of the clients reported that reaching college or university was their highest level of education. At another clinic, however, 14.4% (*n* = 70) of the clients reported that primary school was their highest level of education, and 23.3% (*n* = 113) of the clients reported that reaching college or university was their highest level of education.

Seventh, due to the nature of our MBC system, it was not possible to account for nesting within clinicians. We encountered high staff turnover during the study. For the Implementation Group, this resulted in only 19 clinicians who worked at the organization during the entire implementation period. Although it might be expected that controlling for nesting within clinicians would not change the results, we were unable to confirm this expectation statistically.

Eighth, we provided incentives to the clinics and the clinicians for achieving a specific target (e.g., 80% of the questionnaires completed at the start of treatment or case consultations for more than 50% of the *not-on-track* clients). This may have resulted in external instead of internal motivation, reinforcement of specific behavior instead of real integration of MBC in daily practice, and thus a limited impact on client outcomes.

Last, the number of clinicians in the sample (44 at the start, 42 at the end, and only 19 clinicians at both time points), combined with the aforementioned limitations, makes it clear that the results should be viewed more as exploratory than confirmatory.

### Obstacles to conducting the study

First, we were confronted with low response rates for some outcome measures (11% for discussion of feedback, 28% for change in symptoms, and 27% for satisfaction with treatment), which might have biased the results. This could have been caused by factors at both the clinician and client level. As discussed earlier, it is likely that the clinicians did not strongly encourage their clients to complete the MBC questionnaires because of both an insufficient sense of urgency and external pressures. These factors might have been intensified by client factors, such as a perceived response burden (especially when feedback was not discussed and integrated into the treatment), concerns about the breach of confidentiality, concerns that outcomes might affect the therapeutic relationship, and clients’ reluctance to complete the questionnaires [1, 9].

Second, we were confronted with a consistently lower treatment length in the Implementation Group compared to the Control Group. A possible factor that might have accounted for this difference was the choice for the type of treatment. All of the clinics offered four types of treatment: Short (up to five sessions), Middle (up to eight sessions), Intensive (up to twelve sessions), and Resilience (up to twelve sessions a year). However, the clinicians and clients in the Implementation Group seem to have agreed on Short treatments more often (9.5%, *n* = 803) and Intensive treatments less often (53.1%, *n* = 4,487) then clinicians and clients in the Control Group (5.2%, *n* = 2,461 and 60.4%, *n* = 28,515, respectively). This, in turn, might have resulted from a variety of factors, such as clinicians’ preferences, policies within the clinic, and the municipality's requirements in a specific region.

Third, our MBC implementation team included members who were not experts in MBC nor influencers within their clinics. The members of the implementation team were self-selected, in order to involve clinicians as much as possible and give them control over the improvement of MBC. The premise was that these members would be local champions. Because the MBC results from each clinic and later from each clinician were made visible through the monthly reports, we assumed that clinicians struggling with MBC would go to the local champions for advice. Therefore, we expected that the local champions would also be or would become opinion leaders. However, these assumptions were not always upheld, resulting in a less than optimal MBC implementation team.

Fourth, we were faced with a high rate of staff turnover. This is common when complex evidence-based practices are being implemented [51], but it is a hindrance to the implementation and sustainability of the results [54].

Fifth, for both the Implementation and the Control group, the reported frequency of discussion of feedback and satisfaction with treatment proved to be very high, which could have caused validity issues. The discussion of feedback was based on clients’ response on the CQI, which was part of the MBC questionnaires at the end of the treatment. This means that only clients who completed the questionnaires at the end of treatment rated the extent to which the feedback had been discussed. It is likely that clients who completed the questionnaires at the end of the treatment were motivated to complete these questionnaires because their clinicians had discussed the feedback during treatment. Clients whose clinicians had not discussed the feedback during treatment were probably less motivated to complete the questionnaires at the end of treatment and thus did not indicate a low frequency of discussion of feedback. High scores on both discussion of feedback and satisfaction in the Implementation Group also leave little room for change through the implementation strategies.

Sixth, in the follow-up phase coordination of the maintenance and further improvement of MBC was the responsibility of the local MBC implementation team, with no additional implementation strategies being conducted. The principal members of the local MBC implementation team were also members of the general MBC implementation team. Thus, they had gained experience with a variety of implementation strategies during a longer period of time. We expected, therefore, that the local MBC implementation teams would continue with the activities of the general MBC implementation team. We expected, in fact, that the local MBC implementation teams would have even more impact on the clinicians because more clinicians from each clinic were members of the MBC implementation team, and all members of the MBC implementation team knew all of the clinicians in their clinic. However, these expectations proved to be incorrect, which may have resulted in the questionnaire completion rate decreasing during the follow-up phase to almost the baseline rate. This implies that all of our investment in time, effort, and resources did not sustain MBC after its implementation.

Finally, the results could have been affected by contextual factors [9]. For example, treatment length may have been affected by organizational factors, such as time constraints and high administrative pressures. These factors may make it impossible for clinicians to meet with clients every week and may force clinicians to schedule clients further out. It may be that our implementation strategies had a positive effect on treatment length, but that this effect was negated by the simultaneous increase in organizational pressures that required clinicians to schedule clinical sessions further apart than once per week.

### Comparisons with other studies

Other studies of MBC implementation in adult mental health care are not completely comparable with our study, because most of them appear to have interchanged MBC implementation with the use of it or have intertwined implementation with clinical research [20–22, 24, 55–57]. These studies have reported overall positive effects on use of MBC by clinicians and outcomes for clients. However, some of them have also reported suboptimal implementation [25], a low rate of discussion of the feedback with clients [22, 23], and high rates of staff turnover [20]. Additionally, they have reported that client outcomes improved only when clinicians enhanced their use of MBC [24].

### Strenghts of the study

Despite the results and limitations of our study, we are unaware of any other studies that have used a Type III hybrid design with multifaceted clinician-focused strategies based on an implementation framework to explore the effects of MBC implementation strategies on both clinicians’ uptake and clients’ outcomes. We are also unaware of other studies within general mental health care that have been as large as our study. Our study helps to disentangle the effects of MBC implementation strategies on clinicians’ differential uptake. However, because the self-enforcing loop did not occur, we were unable to explore the effects of MBC implementation strategies on clients’ outcomes.

### Suggestions for future implementation and research

As mentioned above, the results of our study seem to have been limited by (1) clinician factors, (2) insufficient guidance in the underlying implementation framework, (3) the study design, and (4) challenges that arose through the implementation of MBC in a real-world large mental health organization. We faced conceptual challenges regarding the frequency of MBC, how to determine whether the feedback had been discussed with clients, identifying effective incentives, and defining when clinicians should be incentivized. In addition, we faced analytical challenges, such as conducting analyses with imbalanced groups, a high proportion of missing values for some of the outcome measures, and controlling for nested data with a small number of regions. Also, we faced practical challenges, such as a suboptimal MBC implementation team and a high rate of staff turnover. We now provide suggestions for achieving improvement in each of the four domains.

First, it seems essential to take more time to really understand what clinicians want and what they need in MBC implementation and to monitor if those needs are being met, rather than just trying to move forward with the implementation [58]. The Transtheoretical Model (TTM; [59]) explains and predicts an individual's success or failure in achieving a proposed behavior change. Although the TTM was initially applied to changing client behaviors, the TTM can be generalized to organizational change implementation [60]. In accordance with the Transtheoretical Model, clinicians can be classified into the stages Precontemplation, Contemplation, Preparation, Action, and Maintenance. When MBC implementation focuses too much or too quickly on clinicians being in the Action stage, other clinicians will not get on board or the change is not maintained. Organizations and researchers involved in MBC implementation should possibly focus more on clinicians themselves rather than only aim strategies at clinicians. In addition, clinician factors should be measured and controlled for, so that the effects that implementing MBC strategies have on clinicians can be distinguished from the effects that they have on clients.

Second, more guidance is needed for implementing MBC in mental health care and for determining which possibilities can best help to establish the self-enforcing loop. Here, the use of mid-range theories specific to MBC might be helpful [61]. Mid-range theories are limited in scope, less abstract than other theories, address specific phenomena or mechanisms of action, and reflect actual clinical practice. Mid-range theories consist of relatively concrete, operationally defined concepts and propositions that can be tested empirically. Examples of mid-range theories are the Theory of Self-Efficacy [62] and the Theory of Planned Behavior [63]. In addition, we recommend greater use of step-by-step evaluation of each strategy in both process and outcome [64]. Stopping, evaluating, and improving or otherwise changing a specific strategy when needed, might lead to more information about the suitability and potential effects of each strategy.

Third, replication of the current study is needed with more balanced groups, more frequent MBC measurements, shorter questionnaires, different measures of the amount of discussion of feedback and clients’ satisfaction with treatment, a way to account for nesting within clinicians, and incentives for the integration of MBC into daily practice instead of specific behaviors. Also important would be complementary investigation of whether each of the clinician-focused implementation strategies differentially impacts the uptake and outcomes of MBC and whether they may be combined with additional strategies to further enhance results [56].

Last, changes are needed in mental health care organizations that aspire to implement MBC. For example, organizations should facilitate clinicians in their actual use of MBC. This could be done by making available a computerized MBC system that includes measures for both clients and clinicians, staff to support MBC implementation, and time for clinicians to make MBC a part of their daily practice [9]. In addition, organizations should emphasize that the use of MBC is non-negotiable and that experiences with MBC are important for improving both the treatments and the organization as a whole [65]. This could contribute to more frequent measurements being accepted by clinicians and discussed with clients without the use of extrinsic rewards. Also, members of MBC implementation teams should be carefully selected based on the degree to which they are local champions and opinion leaders, so that the effects of MBC implementation strategies can be maximized. To prevent staff turnover from hindering MBC implementation, MBC should be a recurring agenda item at team meetings, and there should be a thorough MBC training protocol for new clinicians to familiarize themselves with the benefits, expectations, and requirements of MBC [54]. These things could improve the effectiveness of MBC implementation, reduce analytical challenges, and eventually contribute to more clear-cut results.

## Conclusions

We have investigated the effects of clinician-focused implementation strategies on clinicians’ uptake of MBC and the resulting outcomes for clients in general mental health care in the Netherlands. We found a significant effect for questionnaire completion rate, but not for the degree to which the feedback was discussed and consequently not for clients’ outcomes. The results appear to have been limited due to clinician factors, insufficient guidance of the underlying implementation framework, study design problems, and obstacles encountered by implementing MBC in a real-world large mental-health care organization. Due to these limitations, the results should be viewed more as exploratory than confirmatory. We are, however, unaware of any other studies of this size that have explored the effects of MBC implementation strategies on clinicians’ uptake and clients’ outcomes within general mental health care. The current study helps to disentangle the effects of MBC implementation strategies on differential uptake by clinicians. Our main suggestion for organizations and researchers that implement MBC is to systematically use specified mid-range theories as a basis for step-by-step implementation and evaluation.

## Data Availability

The datasets that were generated and analyzed during the current study are available from the corresponding author upon request.

## Abbreviations

MBC: Measurement-Based Care
ROM: Routine Outcome Monitoring
FIT: Feedback-Informed Treatment
CFIT: Contextualized Feedback Intervention Theory
TA: Therapeutic Assessment Theory
StaRI: Standards for Reporting Implementation Studies
TREND: Transparent Reporting of Evaluations with Nonrandomized Designs
OQ-45: Outcome Questionnaire-45
CQI: Consumer Quality Index
EPOC: Effective Practice and Organisation of Care taxonomy
RCI: Reliable Change Index
TTM: Transtheoretical Model
